# Supplementary material for: Optimizing the Design of Diatom Biosilica-Targeted Fusion Proteins in Biosensor Construction for Bacillus anthracis Detection
Source: Biology (Basel). 2020 Jan 7;9(1):14. doi: 10.3390/biology9010014 (PMC7168173; doi:10.3390/biology9010014)
Supplement: Supplementary file 1 [file biology-09-00014-s001.pdf]

Optimizing the design of diatom biosilica-targeted fusion proteins in biosensor construction for *Bacillus anthracis* detection

SUPPORTING INFORMATION

Nicole R. Ford [1,5,*], Yijia Xiong[2], Karen A. Hecht [1,4], Thomas C. Squier [2], Gregory L. Rorrer [3], and Guritno Roesijadi [1,3]

1 Marine Biotechnology Group, Pacific Northwest National Laboratory, Sequim, WA 98382, United States
2 Department of Basic Medical Sciences, Western University of Health Sciences, Lebanon, OR 97355, United States
3 School of Chemical Biological and Environmental Engineering, Oregon State University, Corvallis, OR 97331, United States
4 Current Address: AstaReal Inc., 3 Terri Lane, Unit 12, Burlington, NJ 08016, United States
5 Current Address: College of Biosciences, Kansas City University of Medicine and Biosciences, Kansas City, MO 64106, United States

* Correspondence: niford@kcumb.edu

SUPPLEMENTARY METHODS

Diatom-specific destination vector cloning

Restriction cloning was performed to create a new diatom-specific destination vector for fusion protein expression in *T. pseudonana*. The cloning required three steps. Unique restriction sites were added as indicated below.

First, the ER trafficking sequence was added to pTpfcp/*nat* plasmid containing an extra *T. pseudonana fcp* terminator (*fcpT:*pTpfcp/*nat*; an intermediate construct from [1]). Complementary, overlapping olignonucleotides ASN_Sil3ER_F and XSS_Sil3ER_R (see Table S1) were used in an untemplated PCR reaction to create a 98 bp amplicon of the 21 amino acid Sil3 ER trafficking sequence flanked upstream by ApaI, SmaI, and NsiI restriction sites and downstream by XhoI, StuI, and SbfI restriction sites. All six restriction sites are unique in the *fcpT:*pTpfcp/*nat* plasmid sequence. Both the ER trafficking sequence amplicon and the *fcpT:*pTpfcp/*nat* plasmid were digested with ApaI and XhoI (New England Biolabs) and ligated together. The ER trafficking site was now located upstream of the *fcp* terminator.

Second, the *T. pseudonana fcp* promoter was added immediately adjacent to the ER trafficking sequence. The *fcp* promoter was amplified using primers SA_fcpP_F and XZN_fcpP_R (see Table S2). This reaction created a 1006 bp *fcp* promoter amplicon that was flanked upstream by SmaI and ApaI restriction sites and downstream by XhoI, ZraI, and NsiI restriction sites. Both the *fcp* promoter amplicon and plasmid created above were digested with ApaI and NsiI (New England Biolabs) and ligated together. The pTpfcp/*nat* plasmid now contained an additional *fcp* promoter –

ER trafficking sequence – *fcp* terminator cassette, where the *fcp* promoter and ER trafficking sequence were separated only by an XhoI restriction site.

Finally, the tightly coupled *fcp* promoter – ER trafficking sequence were moved into the pDDV1 diatom-specific Gateway destination vector [1]. The *fcp* promoter + ER trafficking sequence cassette was amplified using primers SA_fcpP_F and SA_Sil3ER_Rb (see Table S2). This reaction created a 1067 bp *fcp* promoter amplicon that was flanked upstream by SmaI and ApaI restriction sites and downstream by ApaI and SmaI restriction sites. Both the *fcp* promoter – ER trafficking sequence amplicon and pDDV1[1] were digested with ApaI (New England Biolabs) and ligated together. Presence and orientation of *fcp* promoter – ER trafficking sequence were confirmed by BamHI (New England Biolabs) restriction diagnostic.

Expression clone construction

Invitrogen's Multi-Site Gateway Pro cloning protocol was used to construct entry clones (Tables S2 and S3) and expression clones containing two or three inserts (Table S4) according to manufacturer instructions. All entry and expression clones were verified by DNA sequencing (Genewiz). All diatom expression clones created for this work contain the *T. pseudonana* constitutive *fcp* promoter [2], $Sil3_{T8}$ (a truncated version of the *T. pseudonana* silaffin 3, which has greater silica targeting efficiency than the full-length Sil3 protein [3,4]), the tetracysteine tag for binding AsCy3 [5], V5 and 6xHis epitope tags, and the *T. pseudonana fcp* terminator [2].

Bacterial strains used

All plasmids produced for this work were transformed into and propagated in *E. coli* strain DH5α [F^- φ80*lac*ZΔM15 Δ(*lac*ZYA-*arg*F)U169 *rec*A1 *end*A1 *hsd*R17(r_K^-, m_K^+) *pho*A *sup*E44 λ^- *thi*-1 *gyr*A96 *rel*A1] from Zymo Research.

Expression of the EA1-EGFP antigen was performed in *E. coli* strain T7 Express lysY/Iq [MiniF *lysY lacI*^{q}(Cam^R) / *fhuA2 lacZ::T7 gene1[lon] ompT gal sulA11 R(mcr-73::miniTn10*—Tet^S)*2 [dcm] R(zgb-210::Tn10*—Tet^S) *endA1 Δ(mcrC-mrr) 114::IS10*] from New England Biolabs, as described previously [1].

SUPPLEMENTARY TABLES

Table S1: PCR primers for cloning and analysis

Primer Name	Use[a]	Sequence	Location in Gene
ASN_Sil3ER_F	C	5′-GA**GGGCCC**GGG*ATGCAT*ATGAAGACTTCT GCCATTGTATTGCTTGCCGTTCTCGCCACC-3′	5′ terminus of *T. pseudonana* Sil3 gene ER trafficking sequence (blue) [6]; introduces ApaI (bold), SmaI (underlined), and NsiI (italic) restriction sites; complementary to XSS_Sil3ER_R
XSS_Sil3ER_R	C	5′-**CTCTCGAG**GC*CTGCAGG*AGCGGGGCTCGGT GGCAGCAGTGGTGGCGAGAACGGCAAGCA-3′	3′ terminus of elongated *T. pseudonana* Sil3 gene ER trafficking sequence (blue) [6]; introduces XhoI (bold), StuI (underlined), and SbfI (italic) restriction sites; complementary to XSS_Sil3ER_F
SA_Sil3ER_Rb	C	5′-GGCCC**GGGCCC**GCGGGGCTCGGTGGC AGCAGTGGTGGCGAGAACGGCAAGCA-3′	3′ terminus of elongated *T. pseudonana* Sil3 gene ER trafficking sequence (blue) [6]; introduces ApaI (bold) and SmaI (underlined) restriction sites
SA_fcpP_F	C	5′-GAT**CCCGGG**CCCGCG CTTTTTCCGAGAACTCC-3′	5′ terminus of *T. pseudonana fcp* promoter (green) [2]; introduces SmaI (bold) and ApaI (underlined) restriction sites
XZN_fcpP_R	C	5′-CT**CTCGAG**ACGTC*ATGCATT* TTGGTATCGGTTTGGTAAATC-3′	3′ terminus of *T. pseudonana fcp* promoter (green) [2]; introduces XhoI (bold), ZraI (underlined), and NsiI (italic) restriction sites
fcpP_MP_F[b]	I	5'-GAAGTAACGTATCTTCCCCCTCGACTGGAT-3'	~200 bp upstream of *T. pseudonana fcp* promoter 3' terminus
BA_MP_F[b]	I	5'-CTGATGTGCAGCTGCAGGCGTCTGG-3'	Common 5' terminus of sdAb$_{EA1}$ genes
BA_MP_R[b]	I	5'-TGAGGAGACGGTGACCTGGGTCC-3'	Common 3' terminus of sdAb$_{EA1}$ genes
Sil3_MP_R[b]	I	5'-CTAGACTCCTTTGAGGCCTTGGCATCG	In exon 3 of *T. pseudonana sil3* gene (in T8 fragment)
TpGAPDH_MP_F[b]	I	5'-GGACAAACCGTCACCCACGATAAG-3'	~300 bp downstream of *T. pseudonana* GAPDH gene 5' terminus (Thaps protein ID: 257164)
TpGAPDH_MP_R[b]	I	5'-TGCGTGCACAGATGGTCTCGTA-3'	~200bp upstream of *T. pseudonana* GAPDH gene 3' terminus (Thaps protein ID: 257164)

[a]: C = diatom destination vector, pDDV2, cloning; I = integration verification PCR

[b]: These primers were used in previously published work [1]

Table S2: PCR primers for Gateway entry clones

Primer Name	Sequence[a]
BA_attB1_F	5′-GGGGACAAGTTTGTACAAAAAAGCAGGCTCTGATGTGCAGCTGCAGGCGTCTGG-3′<br>attB1 sdAb$_{EA1}$ [c]
BA_attB5_F	5′-GGGGACAACTTTGTATACAAAAGTTGCTGATGTGCAGCTGCAGGCGTCTGG-3′<br>attB5 sdAb$_{EA1}$ [c]
BA_attB5r_R	5′-GGGGACAACTTTTGTATACAAAGTTGTTGAGGAGACGGTGACCTGGGTCC-3′<br>attB5r sdAb$_{EA1}$ [c]
BA_attB2_R [b]	5′-GGGGACCACTTTGTACAAGAAAGCTGGGTATGAGGAGACGGTGACCTGGGTCC-3′<br>attB2 sdAb$_{EA1}$ [c]
Cy3_T8′_attB5_F	5′-GGGGACAACTTTGTATACAAAAGTTGTGTGTTGTAAGGCTGAGGCTGCCTGTTGCGGTGGAGCTCGTGAGGCCAAATCGAAGCAAGGAAAGACCG-3′<br>attB5 Cy3Tag [d] Sil3$_{T8'}$ [e]
T8′_attB1_F	5′-GGGGACAAGTTTGTACAAAAAAGCAGGCTCTAAATCGAAGCAAGGAAAGACCG-3′<br>attB1 Sil3$_{T8}$
T8_attB2_R	5′-GGGGACCACTTTGTACAAGAAAGCTGGGTACTTCCCACTCTTTCCCTTG-3′<br>attB2 Sil3$_{T8}$
T8′_Cy3_attB5r_R	5′-GGGGACAACTTTTGTATACAAAGTTGTGCAACAGGCAGCCTCAGCCTTACAACAGGCCTCACGAGCTCCACCCTTCCCACTCTTTCCCTTG-3′<br>attB5r Cy3Tag [d] Sil3$_{T8'}$ [e]
ε_EA1_attB1_F	5′-GGGGACAAGTTTGTACAAAAAAGCAGGCTCTTCGATTAACTTAACAAGGAGGTTTCAGCTTATGGGTAAAAGCTTTCCGGA-3′
EA1_attB5r_R[c]	5′-GGGGACAACTTTGTATACAAGTTGTCAGGTTAGGATTATTTAAAACT-3′<br>attB5r *eag* (optimized for *E. coli*)

[a]: Important features are highlighted (green = attB site; blue = amplicon sequence) and annotated as to their identity.
[b]: These primers were used in previously published work [1].
[c]: Since the sdAb$_{EA1}$ nucleotide sequences are identical at their termini, the same primers could be used to amplify both clone G10 and clone A1.
[d]: The binding site for the biarsenical probe AsCy3. See [1] for our previous work with AsCy3. The scope of this short communication, however, does not include work with this small molecule probe.
[e]: Only the silica-targeting peptide is included in Sil3$_{T8'}$ sequence.

Table S3: Gateway entry clones

Entry Clone Name	pDONR	Forward Primer	Reverse Primer	PCR Template	Ref.[a]
pENTR3/2-sdAb$_{EA1}$/G10	pDONR221 P3-P2	BA_attB3_F	BA_attB2_R	pET22B(+)-BA G10	[7]
pENTR1/5r-sdAb$_{EA1}$/G10	pDONR221 P1-P5r	BA_attB1_F	BA_attB5r_R	pET22B(+)-BA G10	[7]
pENTR1/5r-sdAb$_{EA1}$/A1	pDONR221 P1-P5r	BA_attB1_F	BA_attB5r_R	pET22B(+)-BA A1	[7]
pENTR5/2-Cy3Tag-Sil3$_{T8}$′	pDONR221 P5-P2	Cy3_T8′_attB5_F	T8_attB2_R	DDV1-f/T8CEKG	[1]
pENTR1/5r-Sil3$_{T8}$′-Cy3Tag	pDONR221 P1-P5r	T8′_Cy3_attB1_F	T8_attB5r_R	DDV1-f/T8CEKG	[1]
pENTR5/2-sdAb$_{EA1}$/G10	pDONR221 P5-P2	BA_attB5_F	BA_attB2_R	pET22B(+)-BA G10	[7]
pENTR5/2-sdAb$_{EA1}$/A1	pDONR221 P5-P2	BA_attB5_F	BA_attB2_R	pET22B(+)-BA A1	[7]
pENTR1/5r-ε-EA1	pDONR221 P1-P5r	ε_EA1_attB1_F	EA1_attB5r_R	pUC57-eag	[1]

[a]: All citations refer to the first description of the PCR template.

Table S4: Gateway expression clones

Expression Clone Name	Destination Vector	Entry Clone 1	Entry Clone 2	Entry Clone 3	Ref.[a]
pDDV2-sdAb$_{EA1}$/G10-Sil3$_{T8}$′	pDDV2	pENTR1/5r-sdAb$_{EA1}$/G10	pENTR5/2-Cy3Tag-Sil3$_{T8'}$	-	This study
pDDV2-Sil3$_{T8}$′-sdAb$_{EA1}$/G10	pDDV2	pENTR1/5r-Sil3$_{T8}$′-Cy3Tag	pENTR5/2-sdAb$_{EA1/G10}$	-	This study
pDDV1-Sil3$_{T8}$-sdAb$_{EA1}$/G10	pDDV1	pENTR1/4-fcpP [b]	pENTR4r/3r-Sil3T8-Cy3TAG [b]	pENTR3/2-sdAb$_{EA1/G10}$	This study
pDDV2-sdAb$_{EA1}$/A1-Sil3$_{T8}$′	pDDV2	pENTR1/5r-sdAb$_{EA1}$/A1	pENTR5/2-Cy3Tag-Sil3$_{T8'}$	-	This study
pDDV2-Sil3$_{T8}$′-sdAb$_{EA1}$/A1	pDDV2	pENTR1/5r-Sil3$_{T8}$′-Cy3Tag	pENTR5/2-sdAb$_{EA1/A1}$	-	This study
pDDV1-Sil3$_{T8}$-sdAb$_{EA1}$/A1	pDDV1	pENTR1/4-fcpP [b]	pENTR4r/3r-Sil3T8-Cy3TAG [b]	pENTR3/2-sdAb$_{EA1}$ [b]	[1]
pEXP2-ε-EA1-EGFP	pEXP2-DEST	pENTR1/5r-ε-EA1	pENTR5/2-EK-EGFP [b]	-	This study

[a]: All citations refer to the creation of the expression clone.

[b]: These entry clones were created as part of previously published work. See Ref. [1].

▪ SUPPLEMENTARY FIGURES

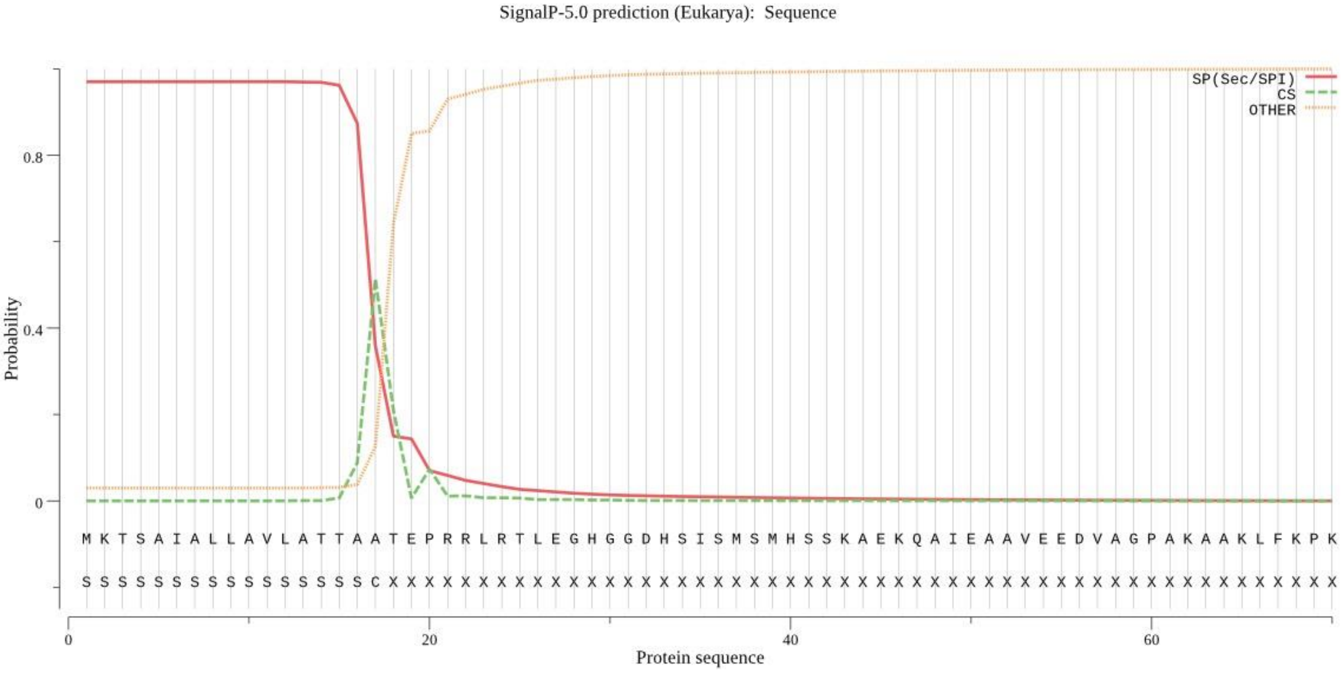

Figure S1. SignalP prediction of ER trafficking sequence. *T. pseudonana* Sil3 amino acid sequence shown along with SignalP-5.0 probability for Sec Signal Peptide (SP) at each amino acid position shown in red, Cleavage Sequence (CS) shown in green, and absence of any kind of signal peptide (Other) shown in yellow. Amino acid positions that are predicted to be part of a signal peptide are denoted "S," the predicted signal sequence cleavage site is denoted "C", and amino acid positions not predicted to form a signal peptide sequence are denoted "X."

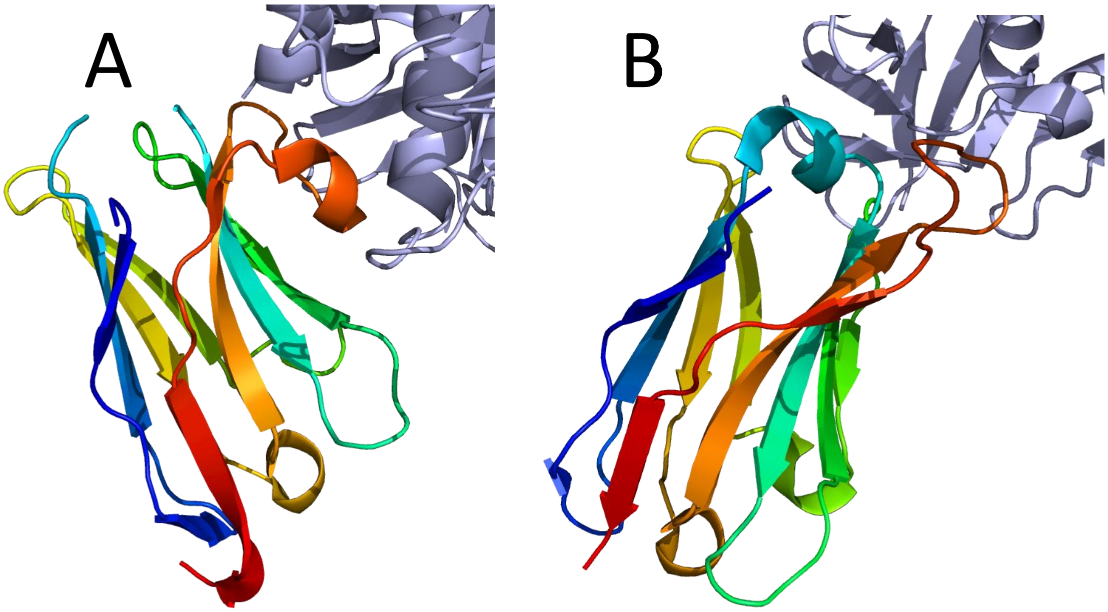

Figure S2. Illustration of two single domain antibodies bound to antigen. In order to confirm homology modeling of antigen binding for sdAb$_{EA1}$/A1 and sdAb$_{EA1}$/G10 presented in Figure 4 of the text, the best matching sdAbs that had available structures with bound antigen are shown. (A) sdAb$_{EA1}$/A1–analogous sdAb (rainbow ribbon structure) bound to Rpn8 and Rpn11 of the 26S proteasome's deubiquitylation module (purple ribbon structure). The antigen binds at the side of the sdAb, away from the N-terminus of the sdAb. (B) sdAb$_{EA1}$/G10–analogous neutralizing sdAb (rainbow ribbon structure) bound to Shiga toxin (purple ribbon structure). The antigen binds at the top of the sdAb, adjacent to the N-terminus of the sdAb. The pdb bank ID of these structures are 4OCN [8] and 4P2C [9], respectively.

- **PEPTIDE SEQUENCES OF FUSION CONSTRUCTS**

> pDDV1-derived Sil3$_{T8}$-sdAb$_{EA1}$/G10

MHPTFLYKVAMKTSAIVLLAVLATTAAGTKSKQGKTEMSVADAKASKESSMPSSKAAKIFKGKSGKGGAREA**CCKAEAACC**TTLYNKVADVQLQASGGGLVQPGGSLKLSCVASGSTFSPDMMRWYRQAPGKQRDLVAWISTSGFTMYADSVKGRFTISRDNAKNTVYLQMNSLKPEDAAVYYCNANRFSGPDYWGQGTGVTVSSYPAFLYKVVDNSKLE**GKPIPNPLLGLDST**RTG**HHHHHH**

> pDDV2-derived Sil3$_{T8}$'-sdAb$_{EA1}$/G10

MHMKTSAIVLLAVLATTAATEPRGPELSTSLYKKAGSKSKQGKTEMSVADAKASKESSMPSSKAAKIFKGKSGKGGAREA**CCKAEAACC**TTLYTKVADVQLQASGGGLVQPGGSLKLSCVASGSTFSPDMMRWYRQAPGKQRDLVAWISTSGFTMYADSVKGRFTISRDNAKNTVYLQMNSLKPEDAAVYYCNANRFSGPDYWGQGTGVTVSSYPAFLYKVVDNSKLE**GKPIPNPLLGLDST**RTG**HHHHHH**

> pDDV2-derived sdAb$_{EA1}$/G10-Sil3$_{T8}$'

MHMKTSAIVLLAVLATTAATEPRGPELSTSLYKKAGSDVQLQASGGGLVQPGGSLKLSCVASGSTFSPDMMRWYRQAPGKQRDLVAWISTSGFTMYADSVKGRFTISRDNAKNTVYLQMNSLKPEDAAVYYCNANRFSGPDYWGQGTGVTVSSTTLYTKVV**CCKAEAACC**GGAREAKSKQGKTEMSVADAKASKESSMPSSKAAKIFKGKSGKYPAFLYKVVDNSKLE**GKPIPNPLLGLDST**RTG**HHHHHH**

> pDDV1-derived Sil3$_{T8}$-sdAb$_{EA1}$/A1

MHPTFLYKVAMKTSAIVLLAVLATTAAGTKSKQGKTEMSVADAKASKESSMPSSKAAKIFKGKSGKGGAREA**CCKAEAACC**TTLYNKVADVQLQASGGGLVQAGGSLELSCVVVGESIIDYQMAWFRQMPVGREREFVAAITGDSHYSDYSASASGRFTISRDNAKKTVSLQMNHLKPEDTALYYCAARKGFGINRLSTAFDYWGHGTGVTVSSYPAFLYKVVDNSKLE**GKPIPNPLLGLDST**RTG**HHHHHH**

> pDDV2-derived Sil3$_{T8}$'-sdAb$_{EA1}$/A1

MHMKTSAIVLLAVLATTAATEPRGPELSTSLYKKAGSKSKQGKTEMSVADAKASKESSMPSSKAAKIFKGKSGKGGAREA**CCKAEAACC**TTLYTKVADVQLQASGGGLVQAGGSLELSCVVVGESIIDYQMAWFRQMPVGREREFVAAITGDSHYSDYSASASGRFTISRDNAKKTVSLQMNHLKPEDTALYYCAARKGFGINRLSTAFDYWGHGTGVTVSSYPAFLYKVVDNSKLE**GKPIPNPLLGLDST**RTG**HHHHHH**

> DDV2-derived sdAb$_{EA1}$/A1-Sil3$_{T8}$'

MHMKTSAIVLLAVLATTAATEPRGPELSTSLYKKAGSDVQLQASGGGLVQAGGSLELSCVVVGESIIDYQMAWFRQMPVGREREFVAAITGDSHYSDYSASASGRFTISRDNAKKTVSLQMNHLKPEDTALYYCAARKGFGINRLSTAFDYWGHGTGVTVSSTTLYTKVV**CCKAEAACC**GGAREAKSKQGKTEMSVADAKASKESSMPSSKAAKIFKGKSGKYPAFLYKVVDNSKLE**GKPIPNPLLGLDST**RTG**HHHHHH**

Color coding corresponds to Figure 1 in the main text: the ER trafficking sequence is shown in orange font, the silica-targeting portion of Sil3$_{T8}$ is shown in green font, sdAb sequences are shown in purple font. AsCy3 binding site as well as V5 and His6 epitope tags are noted in bolded black font.

- **SUPPLEMENTARY REFERENCES**

1. Ford, N.R.; Hecht, K.A.; Hu, D.; Orr, G.; Xiong, Y.; Squier, T.C.; Rorrer, G.L.; Roesijadi, G. Antigen Binding and Site-Directed Labeling of Biosilica-Immobilized Fusion Proteins Expressed in Diatoms. *ACS synthetic biology* **2016**, *5*, 193-199, doi:10.1021/acssynbio.5b00191.
2. Poulsen, N.; Chesley, P.M.; Kröger, N. MOLECULAR GENETIC MANIPULATION OF THE DIATOM THALASSIOSIRA PSEUDONANA (BACILLARIOPHYCEAE)1. *Journal of Phycology* **2006**, *42*, 1059-1065, doi:10.1111/j.1529-8817.2006.00269.x.
3. Sheppard, V.C.; Scheffel, A.; Poulsen, N.; Kroger, N. Live diatom silica immobilization of multimeric and redox-active enzymes. *Applied and environmental microbiology* **2012**, *78*, 211-218, doi:10.1128/aem.06698-11.
4. Poulsen, N.; Scheffel, A.; Sheppard, V.C.; Chesley, P.M.; Kroger, N. Pentalysine clusters mediate silica targeting of silaffins in Thalassiosira pseudonana. *The Journal of biological chemistry* **2013**, *288*, 20100-20109, doi:10.1074/jbc.M113.469379.
5. Cao, H.; Xiong, Y.; Wang, T.; Chen, B.; Squier, T.C.; Mayer, M.U. A red cy3-based biarsenical fluorescent probe targeted to a complementary binding peptide. *Journal of the American Chemical Society* **2007**, *129*, 8672-8673, doi:10.1021/ja070003c.
6. Poulsen, N.; Berne, C.; Spain, J.; Kroger, N. Silica immobilization of an enzyme through genetic engineering of the diatom Thalassiosira pseudonana. *Angewandte Chemie (International ed. in English)* **2007**, *46*, 1843-1846, doi:10.1002/anie.200603928.
7. Walper, S.A.; Anderson, G.P.; Brozozog Lee, P.A.; Glaven, R.H.; Liu, J.L.; Bernstein, R.D.; Zabetakis, D.; Johnson, L.; Czarnecki, J.M.; Goldman, E.R. Rugged single domain antibody detection elements for Bacillus anthracis spores and vegetative cells. *PloS one* **2012**, *7*, e32801, doi:10.1371/journal.pone.0032801.
8. Pathare, G.R.; Nagy, I.; Sledz, P.; Anderson, D.J.; Zhou, H.J.; Pardon, E.; Steyaert, J.; Forster, F.; Bracher, A.; Baumeister, W. Crystal structure of the proteasomal deubiquitylation module Rpn8-Rpn11. *Proceedings of the National Academy of Sciences of the United States of America* **2014**, *111*, 2984-2989, doi:10.1073/pnas.1400546111.
9. Lo, A.W.; Moonens, K.; De Kerpel, M.; Brys, L.; Pardon, E.; Remaut, H.; De Greve, H. The molecular mechanism of Shiga toxin Stx2e neutralization by a single-domain antibody targeting the cell receptor-binding domain. *The Journal of biological chemistry* **2014**, *289*, 25374-25381, doi:10.1074/jbc.M114.566257.
